# The effect of word spacing size on word segmentation in Tibetan reading: the moderating role of Tibetan proficiency

**DOI:** 10.3389/fpsyg.2026.1922806

**Published:** 2026-09-11

**Authors:** Chenxu Zhang, Hongyu Liang, Zijian Xie, Xiaolei Gao, Lei Gao

**Affiliations:** Plateau Brain Science Research Center, Xizang University, Lhasa, China

**Keywords:** eye movements, Tibetan proficiency, Tibetan reading, word segmentation, word spacing size

## Abstract

**Introduction:**

Previous studies have shown that inserting interword spaces facilitates word recognition, reading efficiency, and saccadetarget selection during Tibetan reading. However, the effects of different wordspacing sizes on Tibetan reading remain unclear. Therefore, this study investigated the effect of wordspacing size on word segmentation during Tibetan reading and examined the moderating role of Tibetan proficiency.

**Methods:**

This study adopted a 2 (Tibetan proficiency: high vs. low) × 3 (word spacing size: normal, half-width spaced, full-width spaced) mixed experimental design. A total of 67 students recruited from Xizang University participated in the experiment. An eyeLink 1000 Plus eye tracker was used to record their eye movement data while reading Tibetan sentences presented with different word spacing sizes.

**Results:**

The results showed that: (1) interword spacing facilitated Tibetan text reading and lexical recognition, with the facilitative effect increasing as spacing size increased; (2) Tibetan proficiency moderated the spacing effect. Specifically, full-width spaces provided a stronger facilitative effect on lexical recognition for low-proficiency readers.

**Discussion:**

These findings suggest that increased word spacing benefits Tibetan reading, particularly among readers with lower Tibetan proficiency.

## 1 Introduction

Reading is a crucial and complex cognitive activity, serving as one of the primary avenues for knowledge acquisition in modern society (Bai et al., 2017; Liu et al., 2019). It is a meaning-construction process that begins with character and lexical recognition and culminates in the comprehension of discourse meaning (Xiang et al., 2018). Lexical recognition is a critical stage of reading. It requires readers to segment continuous visual input into linguistic units, such as words, phrases, and clauses, and to determine the relationships among these units. This process is referred to as word segmentation (Li and Rayner, 2009; Liu et al., 2013; Ma and Li, 2014; Rayner and Fischer, 1998). As the primary stage of lexical processing, word segmentation contributes significantly to both information processing and cognitive mechanisms during reading (Li and Rayner, 2009; Rayner, 2009). How can efficient and accurate word segmentation be achieved? Clear visual word segmentation cues undoubtedly serve a critical function.

In most alphabetic writing systems, interword spaces serve as vital visual word segmentation cues. In English, these spaces guide the reader's gaze positioning, facilitate lexical recognition, and shorten reading time; if these spaces are removed or replaced with other forms, such as random digits or letter-like fillers, English reading efficiency significantly decreases (Epelboim and Booth, 1994; Malt, 1978; O'Regan, 1992; O'Regan et al., 1984; Pollatsek, 1982; Sheridan and Rayner, 2013; Slattery, 2013). A similar phenomenon is observed in Spanish, another alphabetic writing system with interword spaces; specifically, when these spaces are removed or replaced, reading speed in Spanish declines significantly (Perea, 2009).

Unlike alphabetic writing systems, languages such as Thai and Japanese lack interword spaces as word segmentation cues. How does inserting interword spaces affect the reading of these scripts? In Thai, adding spaces improved reading speed and accuracy in a reading-aloud task (Kohsom, 1997). Eye-tracking evidence further indicated that spaces facilitate target-word processing, as reflected in shorter gaze durations, but did not improve sentence-level reading time or initial saccade landing position (Winskel and Radach, 2009). Even without spaces, Thai readers appear to use the frequencies of word-initial and word-final characters to infer likely word boundaries and guide saccade targeting. The frequency of character pairs that occur across word boundaries provides an indirect measure of word boundary information (Kasisopa et al., 2013). Research on Japanese has shown that interword spacing facilitates word identification and eye-movement guidance in text written entirely in Hiragana, a phonographic syllabary. In contrast, spacing does not facilitate reading in mixed Kanji-Hiragana text, because the visually salient Kanji characters already provide effective segmentation cues. These findings suggest that interword spacing is particularly beneficial when a writing system provides relatively limited visual cues to word boundaries (Sainio et al., 2007).

Similarly, Chinese text lacks visual word segmentation cues such as interword spaces. Then, how does inserting interword spaces affect Chinese reading? Some studies have found that interword spaces hinder Chinese reading (Liu et al., 1974). Other research, however, has suggested that such spaces facilitate the reading process (Hsu, 2000; Wang, 2015). Nevertheless, a larger body of research indicates that interword spaces neither hinder nor facilitate Chinese reading overall, but they can promote lexical recognition (Bai et al., 2008; Ma, 2017; Ma and Li, 2019; Zang et al., 2013).

In summary, existing research on interword spaces has largely been confined to the single dimension of “presence vs. absence,” primarily exploring how the presence of spaces affects reading processing, word identification, and saccade targeting. This unidimensional focus has a significant limitation: it overlooks the critical variable of “space size.” Previous research has pointed out that the physical size of a space may be the core variable moderating reading effects (Slattery, 2013), a claim supported by evidence from Chinese reading research. Empirical findings have demonstrated that when interword spaces were either half-character or full-character in width, participants' reading times were shorter than in the normal unspaced condition; notably, the accuracy of answering comprehension questions was significantly higher in the half-character space condition than in the full-character space condition. This suggests that the optimal interword interval for Chinese text may lie between traditional spacing and a full-character space (Hsu, 2000). To further clarify the optimal size of Chinese interword spaces, Liang (2010) manipulated space size and presentation format. They found that half-character spaced text did not differ significantly from unspaced text on multiple eye-movement measures, including total fixation duration and reading rate; however, compared with full-character spacing, half-character spacing yielded better performance on indicators such as total number of fixations and reading rate. Based on these results, the authors concluded that, under the conditions of their study, half-character spacing neither facilitates nor interferes with Chinese reading, whereas full-character spacing disrupts it.

Tibetan belongs to the Sino-Tibetan language family (Bai et al., 2017; Gao X. L. et al., 2024) and employs an abugida writing system. Specifically, a Tibetan written syllable is organized around a root consonant. The root consonant may occur alone with an inherent vowel, and additional consonant letters may be attached as prefixes or suffixes, or stacked vertically to form a spatial structure. Crucially, Tibetan text normally contains no interword spaces; instead, syllables are separated by the delimiter known as the “▾ ” (tsek). While this delimiter aids in syllable segmentation, it does not provide reliable information about word boundaries, so word boundaries in Tibetan text are ambiguous (Wang et al., 2024). Although studies have shown that manually inserting interword spaces significantly facilitates word recognition, improves reading efficiency, and optimizes saccade target selection in Tibetan reading (Wang et al., 2024), the mechanism underlying the core physical variable-word spacing size-remains unclear. Specifically, do spaces of different sizes exert differential effects? Is the pattern of influence consistent with that observed in Chinese, or does it show unique characteristics due to Tibetan's distinctive orthographic structure? These are the key questions the present study aims to address.

The interword spacing effect in native Tibetan reading may be moderated by readers' Tibetan language proficiency. Support for this comes from native Chinese reading: both Shen et al. (2010) and Yan et al. (2012) found that nonword spacing disrupted reading more severely for less proficient than for skilled readers, indicating their greater reliance on external word-boundary cues. A similar proficiency-dependent pattern is observed in Chinese as a second language. Specifically, Everson (1986) found that artificial spaces mainly impaired the strategies of advanced learners with minimal effects on beginners, whereas Han and Jiang (2018) demonstrated that interword spacing facilitated reading among Korean learners, with less proficient readers exhibiting greater reductions in reading times and fixation counts. In summary, these findings suggest that language proficiency modulates readers' dependence on explicit word-segmentation cues, supporting Tibetan language proficiency as a moderator of this effect.

Therefore, the present study recruited students from Xizang University as participants to investigate the effects of word spacing size on word segmentation during Tibetan reading. Simultaneously, by manipulating participants' Tibetan proficiency, this study examined the moderating role of Tibetan proficiency in the spacing effect. The present study aimed to address two core questions: (1) The effect of word spacing size on word segmentation in Tibetan reading, leading to Hypothesis 1: interword spaces facilitate Tibetan reading, and this facilitative effect increases as the space size expands. (2) The moderating role of Tibetan proficiency in word segmentation, leading to Hypothesis 2: Tibetan proficiency moderates the spacing effect; specifically, full-width spaces provide a stronger facilitative effect on lexical recognition for low-proficiency readers.

## 2 Materials and methods

### 2.1 Experimental design

The experiment adopted a 2 (Tibetan proficiency: high, low) × 3 (word spacing size: normal, half-width spaced, full-width spaced) two-factor mixed experimental design. Among these variables, Tibetan proficiency served as the between-subjects variable, while word spacing size was the within-subjects variable. The normal condition followed the standard Tibetan writing format: all tseks were retained and no extra spaces were added. In the half-width-spaced and full-width-spaced conditions, interword tseks were replaced by half-width or full-width spaces, respectively, while intraword tseks remained unchanged.

### 2.2 Participants and ethics

Based on recommendations for power analysis in mixed designs (Cohen, 1988; Faul et al., 2009), the statistical power was set at 0.8, with a medium effect size (*f* = 0.25) and a correlation coefficient of 0.5 among repeated measures. Calculations using G^*^Power 3.1.9.7 (Heinrich Heine University Düsseldorf, Düsseldorf, Germany) indicated that a minimum of 28 participants was required, with 14 per group. Considering the potential for invalid data, 67 students (17 males, 50 females) were recruited from Xizang University, with a mean age of *M* = 20.1 years (SD = 1.12). Participants' Tibetan proficiency grouping was based on their scores on the Tibetan-language component of the National College Entrance Examination (NCEE; maximum score = 150), a standardized examination assessing grammar, reading comprehension, and writing. Thus, the scores reflect individual differences in written Tibetan ability among native Tibetan speakers. Participants scoring ≥140 were classified as high-proficiency readers (*n* = 36, *M* = 140.97, SD = 1.65), whereas those scoring ≤ 110 were classified as low-proficiency readers (*n* = 31, *M* = 103.97, SD = 6.63). All participants were native Tibetan speakers and reported Tibetan as their first language (L1). All participants had normal or corrected-to-normal vision, no history of physical or mental illness or reading disabilities, and were right-handed. The experiment was approved by the Ethics Committee of Xizang University (IRB NO: XZDXLL2022016). All participants provided written informed consent, participated voluntarily, and received compensation after the experiment.

### 2.3 Experimental materials

Drawing on previous literature (Wang et al., 2023), 120 Tibetan declarative sentences were developed based on university-level Tibetan textbooks and extracurricular reading materials of comparable difficulty, with appropriate adaptations. All sentences were free of syntactic or semantic ambiguity, with lengths controlled between 25 and 30 Tibetan character units. After the sentences were compiled, 16 Tibetan college students were invited to rate the difficulty and fluency of the sentences using a 5-point Likert scale. The results were as follows: Difficulty (*M* = 1.80, SD = 0.92), where 1 represents “very simple”, Fluency (*M* = 2.10, SD = 1.15), where 1 represents “very fluent.”

Furthermore, word segmentation was performed on these sentences according to the standards of the “*Modern Tibetan Frequency Dictionary*” (Lu and Gao, 2007). The same 16 Tibetan college students evaluated the consistency of the word segmentation, which reached 86.03%. These assessment results indicated that all 120 candidate Tibetan sentences met the experimental requirements. After further balancing for sentence length, 90 sentences were finally selected for the experiment (*M* = 26.97, SD = 2.06 in length), comprising 84 formal experimental sentences and six practice sentences. The students who participated in the assessment of difficulty, fluency, and word segmentation consistency did not participate in the subsequent formal experiment.

As shown in Table 1, experimental sentences were presented under three conditions in the formal experiment, with each condition consisting of 28 formal experimental sentences and two practice sentences. A Latin square design was employed to determine the presentation order of the sentences across conditions, resulting in the construction of three blocks. Each participant read only one experimental sequence, namely one block. Furthermore, to ensure that participants read carefully and understood the meaning of the sentences, a reading comprehension question followed one-third of the formal experimental sentences. Participants were required to make a “Yes” or “No” judgment, with half of the correct answers being “Yes” and the other half being “No”.

**Table 1:** Examples of experimental materials under three presentation conditions.

Word spacing size	Examples
Normal	སློབ་མས་བོད་ཀྱི་དེབ་གསར་པ་བཀླགས་པས་ཤེས་ཡོན་རྒྱས།
Half-width spaced	སློབ་མས བོད་ཀྱི དེབ གསར་པ བཀླགས་པས ཤེས་ཡོན རྒྱས།
Full-width spaced	སློབ་མས བོད་ཀྱི དེབ གསར་པ བཀླགས་པས ཤེས་ཡོན རྒྱས།

Note: The above sentence means: Students gained knowledge by reading new Tibetan-language books.

### 2.4 Experimental apparatus

The experimental apparatus was an EyeLink 1000Plus eye tracker (SR Research, Canada) with a sampling frequency of 1000 Hz. Data collection utilized the default settings for cognitive research on the EyeLink 1000 Plus: a saccade velocity threshold of 30°/sec, a saccade acceleration threshold of 8000°/sec^2^, and a saccade motion threshold of 0.1° (Liao and Li, 2022; Wang et al., 2023). The stimuli were presented on a 24.5-inch DELL monitor (Dell Inc., Round Rock, TX, USA) with a screen refresh rate of 240 Hz and a resolution of 1920 × 1080 pixels. Following previous literature (Li et al., 2022), the materials were displayed in Microsoft Himalaya font at a size of 36 pt. The distance between the participants' eyes and the screen was approximately 65 cm. Each Tibetan character subtended a visual angle of approximately 0.6°.

### 2.5 Experimental procedure

**Figure 1:**
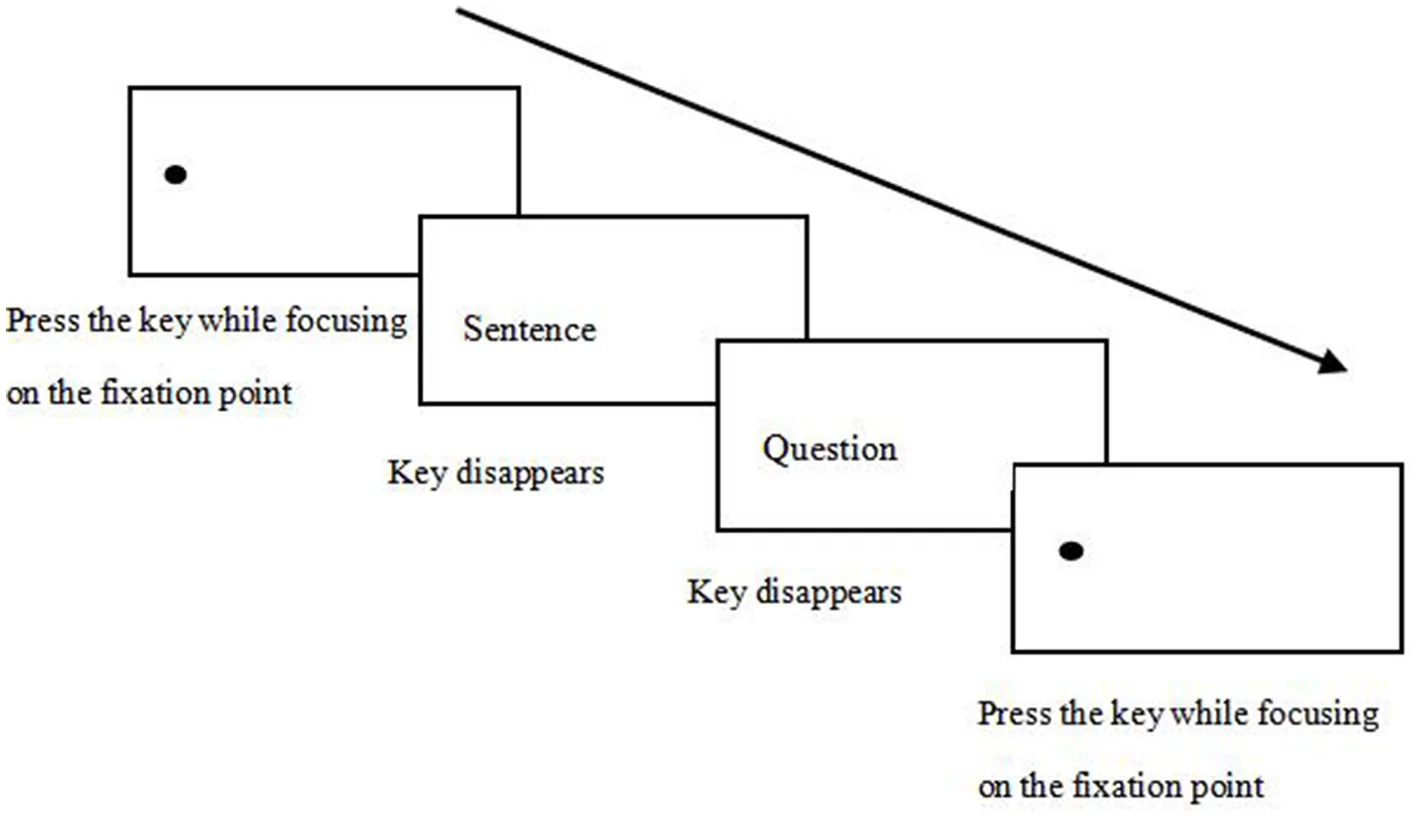
The flowchart of the experimental procedure.

The experiment was conducted individually, following these specific procedures:(1) Preparation: upon entering the laboratory, the participant sat in the designated seat and placed their head in the correct position required for the experiment. The experimenter instructed the participant to remain as still as possible throughout the process. (2) Instructions: the participant began by silently reading the instructions on the screen. Once they indicated they had finished reading, the experimenter provided a brief summary and emphasized that Tibetan sentences would be presented one by one. The participant was instructed to read silently at their normal pace. They were also informed of the locations of the “Page Down” key and the “Yes/No” judgment keys and were instructed to press the “Page Down” key to start reading the next sentence while focusing on the fixation point on the left side of the screen. (3) Calibration: a three-point calibration was employed, with the error value controlled below 0.25. (4) Practice Phase: following calibration, a practice phase was conducted. During this stage, the participant read six sentences (two for each of the three conditions) to become familiar with the experimental process and operational requirements. (5) Formal Experiment: after the practice phase, the formal experiment commenced. The environment remained quiet with constant lighting throughout the session, and participants were allowed to request a break if needed. The total time for each participant to complete the entire experiment was approximately 30–45 min. The experimental flow is illustrated in Figure 1.

### 2.6 Experiment indicators

Drawing on previous literature (Bai et al., 2011; Ma and Li, 2019; Wang et al., 2020; Yan et al., 2013), we selected the average fixation duration, average saccade amplitude, total number of fixations, forward saccade amplitude, and total sentence reading time as global analysis indicators. Specifically, the average fixation duration refers to the average duration of all fixations within a sentence, which reflects the difficulty of sentence processing. The average saccade amplitude refers to the average distance moved by the eyes between two adjacent fixations, reflecting the efficiency of information acquisition. The total number of fixations represents the total fixation count within a sentence, reflecting the overall cognitive processing load, where a higher load results in a greater number of fixations. The forward saccade amplitude is the average distance of forward eye movements, which specifically serves as an indicator of reading fluency during sentence processing. Finally, the total sentence reading time refers to the time from the beginning to the end of sentence reading, comprehensively reflecting the overall cognitive load of sentence processing, including all stages such as early visual encoding, word identification, semantic integration, and regressive eye movements.

**Figure 2:**
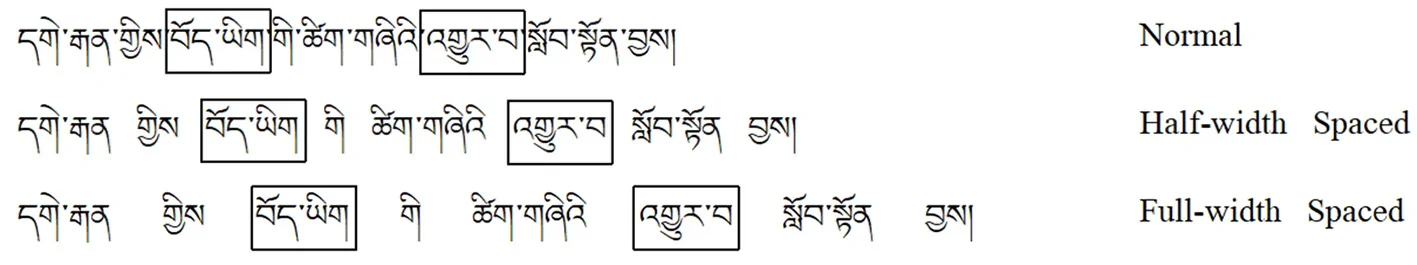
An illustration of local analysis based on areas of interest in different conditions (the boxes mean areas of interest). Translation: the teacher taught the inflectional changes of Tibetan word roots.

Drawing on previous literature (Ma and Li, 2019; Rayner, 1998; Wang et al., 2024; Yan et al., 2013), we conducted local analysis for all three spacing conditions. For each experimental sentence, two interest areas (IAs) were selected for word-based local analyses, each consisting of one two-character word spanning four horizontal letter-space units. The IAs were identical across the three spacing conditions in terms of physical size, textual content, and spatial distribution, thereby ensuring comparability of local processing measures. To minimize potential interference from fixations associated with the onset and offset of reading, the IAs were positioned away from both the beginning and the end of the sentence. The interest areas for local analysis are illustrated in Figure 2. Among the local analysis indicators, the regressive duration refers to the total duration of all fixations occurring during regressions back to the current interest area. The gaze duration refers to the sum of the fixation duration on an interest area from the moment the eyes first enter the area until they first leave it. The total number of fixations refers to the total count of all fixations falling within the interest area. The total fixation duration refers to the sum of all fixation durations falling within the interest area, including regression time. Specifically, the gaze duration is an indicator reflecting early-stage lexical processing efficiency, while regressive duration, total number of fixations, and total fixation duration are indicators reflecting late-stage lexical processing efficiency.

## 3 Results

### 3.1 Data processing

Every participant in both groups achieved a comprehension accuracy above 80%, with an overall mean of 87.59% and no significant difference between high-proficiency (88.62%) and low-proficiency readers (86.40%), *t*(65) = 1.74, *p* >0.05, indicating that all participants read the sentences carefully and understood the content. To further ensure the accuracy of the data analysis, and following previous studies (Bai et al., 2008, 2011; Wang et al., 2024), raw data were excluded based on the following criteria: (1) fixation durations shorter than 80 ms or longer than 1200 ms; (2) experimental interruptions caused by premature or incorrect key presses; (3) invalid data lost during the eye-tracking process; (4) sentences with fewer than four fixations; (5) data points falling outside three standard deviations from the mean. Data excluded from the global analysis accounted for 2.41% of the total data, while data excluded from the local analysis accounted for 1.46%. Additionally, practice sentences were used only to familiarize participants with the experimental procedure, and their data were not included in the subsequent analyses.

Data analysis was conducted in the R environment (R Core Team, 2021) using linear mixed-effects models (LMMs) with the lme4 package. LMMs allow for all remaining valid data to be included in the statistical analysis, thereby improving data utilization. Before fitting the linear mixed-effects models, a logarithmic transformation was applied to the eye-movement measures to meet the model assumptions. Specifically, the average fixation duration, average saccade amplitude, total number of fixations, forward saccade amplitude, total sentence reading time, regressive duration, gaze duration, and total fixation duration were analyzed using linear mixed-effects models as continuous variables (Baayen and Davidson, 2008; Wang et al., 2024). Word spacing size and Tibetan proficiency were included as fixed factors, while the crossed random effects of participants and items were included as random factors to enhance the stability of the statistical results. An absolute value of t >1.96 at the 5% level indicates significance.

### 3.2 Data analysis

#### 3.2.1 Global analysis

**Figure 3:**
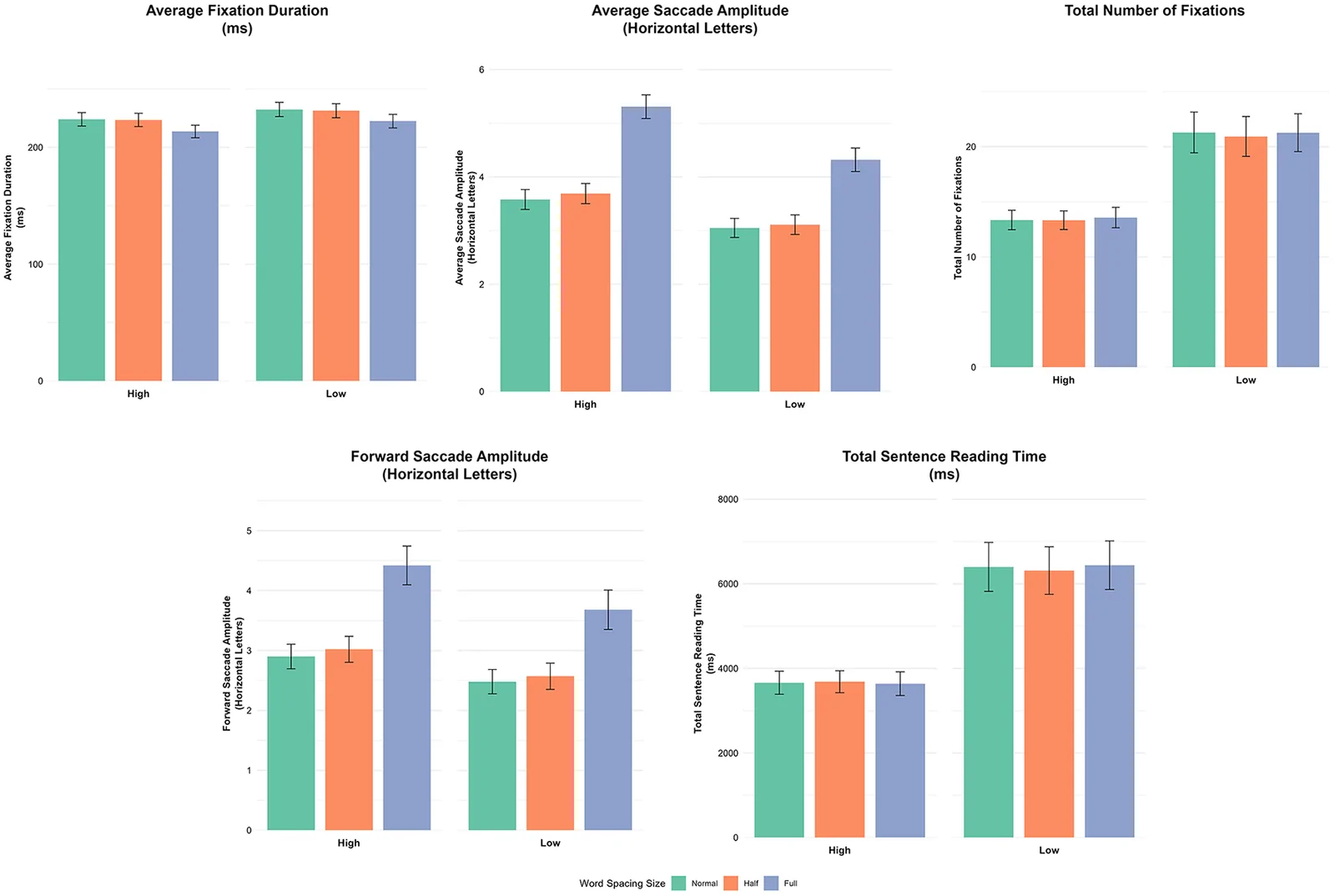
Bar charts of eye movement measures across conditions in the global analysis.

The descriptive statistics for each eye-movement indicator under different word spacing conditions are presented in Table 2 and Figure 3, and the results of the statistical analysis are shown in Table 3.

**Table 2:** Means and standard deviations of global eye-movement indicators.

Word spacing size	Tibetan proficiency	Average fixation duration (ms)	Average saccade amplitude (horizontal letters)	Total number of fixations	Forward saccade amplitude (horizontal letters)	Total sentence reading time(ms)
Normal	High proficiency	224.02 (34.44)	3.58 (1.11)	13.35 (5.30)	2.90 (1.23)	3661.66 (1635.69)
	Low proficiency	232.46 (33.92)	3.05 (0.99)	21.29 (10.30)	2.48 (1.13)	6490.09 (3221.92)
Half-width spaced	High proficiency	223.49 (34.00)	3.69 (1.13)	13.33 (5.07)	3.02 (1.30)	3684.78 (1559.05)
	Low proficiency	231.37 (33.37)	3.11 (1.02)	20.93 (10.06)	2.57 (1.22)	6314.43 (3127.01)
Full-width spaced	High proficiency	213.59 (32.36)	5.31 (1.32)	13.57 (5.57)	4.42 (1.94)	3639.15 (1683.17)
	Low proficiency	222.44 (32.32)	4.32 (1.22)	21.27 (9.57)	3.68 (1.83)	6440.75 (3199.41)

**Table 3:** Statistical Analysis results of global eye-movement indicators.

Effects and contrasts	Statistic	Average fixation duration (ms)	Average saccade amplitude (horizontal letters)	Total number of fixations	Forward saccade amplitude (horizontal letters)	Total sentence reading time(ms)
Tibetan proficiency	*b*	−0.04	0.17	−0.39	0.18	−0.55
	SE	0.03	0.04	0.07	0.04	0.08
	*t*	−1.61	4.46^***^	−5.37^***^	4.87^***^	−6.59^***^
Space: half-width vs. normal	*b*	−0.01	0.03	−0.01	0.03	−0.02
	SE	0.01	0.01	0.01	0.01	0.01
	*t*	−0.91	2.19^*^	−0.48	5.49^***^	−1.22
Space: full-width vs. normal	*b*	−0.05	0.38	0.03	0.36	0.01
	SE	0.01	0.01	0.01	0.01	0.01
	*t*	−8.08^***^	27.79^***^	2.31^*^	61.94^***^	0.11
Space: half-width vs. full-width	*b*	0.04	−0.35	−0.04	−0.33	−0.02
	SE	0.01	0.01	0.01	0.01	0.01
	*t*	7.19^***^	−25.69^***^	−2.79^*^	−56.20^***^	−1.34
Proficiency × space: half-width vs. normal	*b*	0.01	0.01	0.01	0.01	0.03
	SE	0.01	0.02	0.02	0.01	0.02
	*t*	0.50	0.41	0.65	0.32	1.63
Proficiency × space: full-width vs. normal	*b*	−0.01	0.05	−0.02	0.04	−0.01
	SE	0.01	0.02	0.02	0.01	0.01
	*t*	−0.25	2.59^**^	−0.93	4.66^***^	−0.42
Proficiency × space: half-width vs. full-width	*b*	0.01	−0.04	0.03	−0.04	0.04
	SE	0.01	0.02	0.02	0.01	0.02
	*t*	0.76	−2.18^*^	1.58	−4.33^***^	2.06^*^

Note. ^***^ *p* < 0.001, ^**^ *p* < 0.01, ^*^ *p* < 0.05.

The main effect of Tibetan proficiency was significant. Specifically, compared to the low-proficiency group, the high-proficiency group exhibited larger average saccade amplitude (*b* = 0.17, SE = 0.04, *t* = 4.46, *p* < 0.001) and larger forward saccade amplitude (*b* = 0.18, SE = 0.04, *t* = 4.87, *p* < 0.001). Furthermore, the high-proficiency group showed fewer total fixations (*b* = −0.39, SE = 0.07, *t* = −5.37, *p* < 0.001) and shorter total sentence reading time (*b* = −0.55, SE = 0.08, *t* = −6.59, *p* < 0.001).

The main effect of word spacing size was significant. Specifically, compared to the normal condition, the half-width spaced condition exhibited larger average saccade amplitude (*b* = 0.03, SE = 0.01, *t* = 2.19, *p* < 0.05) and larger forward saccade amplitude (*b* = 0.03, SE = 0.01, *t* = 5.49, *p* < 0.001). Compared to the normal condition, the full-width spaced condition showed shorter average fixation duration (*b* = −0.05, SE = 0.01, *t* = −8.08, *p* < 0.001), larger average saccade amplitude (*b* = 0.38, SE = 0.01, *t* = 27.79, *p* < 0.001), larger forward saccade amplitude (*b* = 0.36, SE = 0.01, *t* = 61.94, *p* < 0.001), and a greater number of fixations (*b* = 0.03, SE = 0.01, *t* = 2.31, *p* < 0.05). Furthermore, compared to the full-width spaced condition, the half-width spaced condition resulted in longer average fixation duration (*b* = 0.04, *SE* = 0.01, *t* = 7.19, *p* < 0.001), smaller average saccade amplitude (*b* = −0.35, SE = 0.01, *t* = −25.69, *p* < 0.001), smaller forward saccade amplitude (*b* = −0.33, SE = 0.01, *t* = −56.20, *p* < 0.001), and fewer fixations (*b* = −0.04, SE = 0.01, *t* = −2.79, *p* < 0.05). Taken together, although full-width spaces led to an increase in the number of fixations due to the increased physical length of the text, their facilitative effects in shortening average fixation duration and increasing saccade amplitude were significantly superior to both the half-width spaced and normal conditions.

The interaction between Tibetan proficiency and word spacing size was significant. Specifically, in the contrast between full-width spaced and normal conditions, a significant interaction was observed in average saccade amplitude (*b* = 0.05, SE = 0.02, *t* = 2.59, *p* < 0.01) and forward saccade amplitude (*b* = 0.04, *SE* = 0.01, *t* = 4.66, *p* < 0.001). In the contrast between half-width spaced and full-width spaced conditions, significant interactions were found in average saccade amplitude (*b* = −0.04, SE = 0.02, *t* = −2.18, *p* < 0.05), forward saccade amplitude (*b* = −0.04, SE = 0.01, *t* = −4.33, *p* < 0.01), and total reading time (*b* = 0.04, SE = 0.02, *t* = 2.06, *p* < 0.05). These results indicate that the effect of word spacing size on the reading process is significantly modulated by Tibetan proficiency. Further simple effect analysis revealed that for the high-proficiency group, compared to the normal condition, the full-width spaced condition resulted in larger average saccade amplitude (*b* = 0.41, SE = 0.01, *t* = 35.71, *p* < 0.001) and larger forward saccade amplitude (*b* = 0.40, SE = 0.01, *t* = 62.29, *p* < 0.001). Compared to the half-width spaced condition, the full-width spaced condition also showed larger average saccade amplitude (*b* = 0.38, SE = 0.01, *t* = 32.96, *p* < 0.001) and larger forward saccade amplitude (*b* = 0.37, SE = 0.01, *t* = 56.92, *p* < 0.001). For total sentence reading time, pairwise differences among the three conditions did not reach statistical significance (*p*s > 0.05). For the low-proficiency group, compared to the normal condition, the full-width spaced condition exhibited larger average saccade amplitude (*b* = 0.36, SE = 0.01, *t* = 27.75, *p* < 0.001) and larger forward saccade amplitude (*b* = 0.36, SE = 0.01, *t* = 61.94, *p* < 0.001). Compared to the half-width spaced, the full-width spaced condition resulted in larger average saccade amplitude (*b* = 0.34, SE = 0.01, *t* = 26.25, *p* < 0.001) and larger forward saccade amplitude (*b* = 0.33, SE = 0.01, *t* = 56.20, *p* < 0.001). For total sentence reading time, pairwise differences among the three conditions also failed to reach significance (*p*s > 0.05).

Global analysis revealed that the average saccade amplitude and forward saccade amplitude were larger in the half-width spaced condition than in the normal condition. Furthermore, compared to both the normal and half-width spaced conditions, the full-width spaced condition resulted in shorter average fixation duration and even larger average and forward saccade amplitudes. These findings indicate that inter-word spacing exerts a facilitative effect on the reading of Tibetan text, and this facilitative effect increases as the spacing size increases.

#### 3.2.2 Local analysis

**Figure 4:**
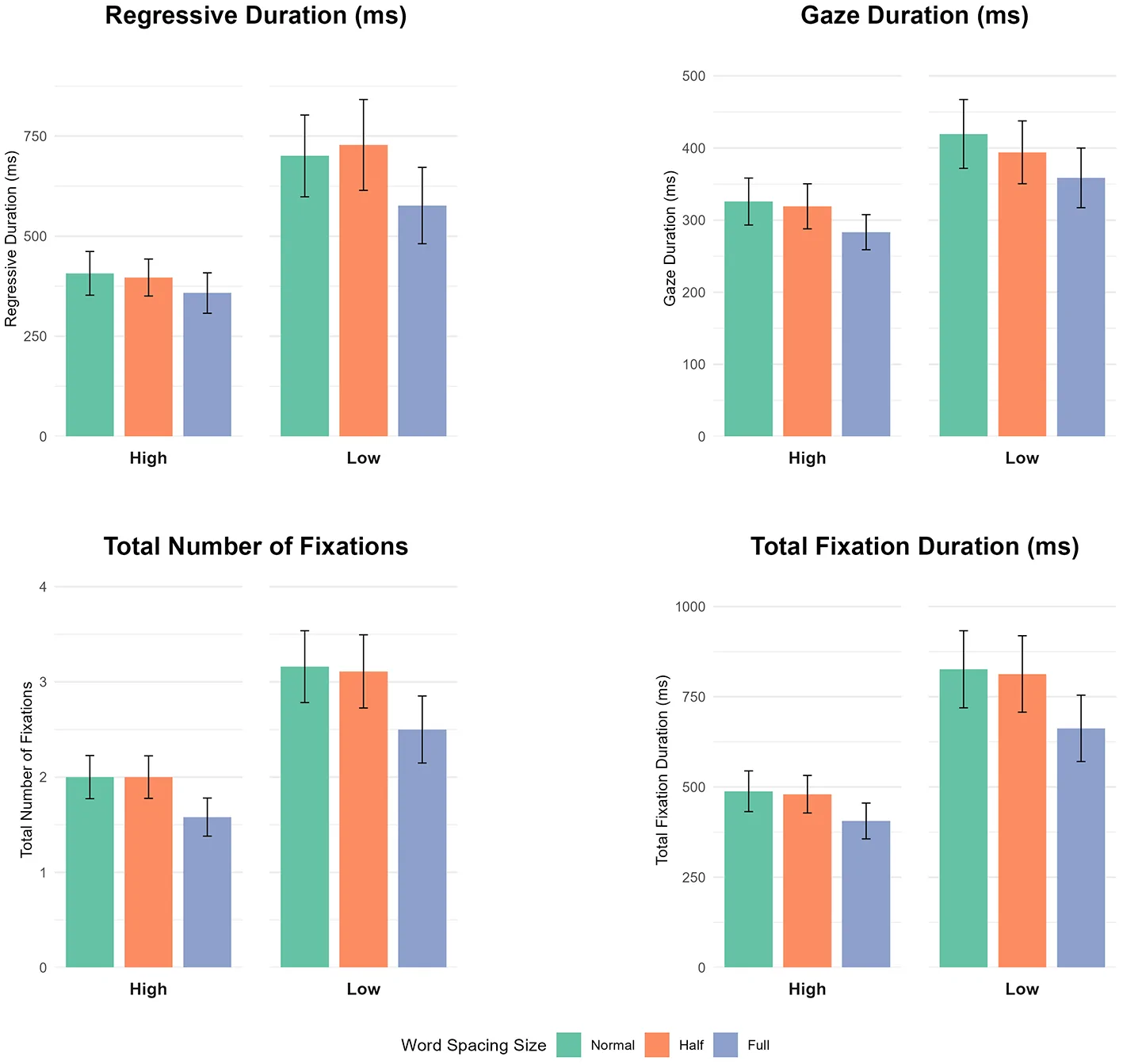
Bar charts of eye movement measures across conditions in the local analysis.

The descriptive statistics for each eye-movement indicator under different word spacing conditions are presented in Table 4 and Figure 4, and the results of the statistical analysis are shown in Table 5.

**Table 4:** Means and standard deviations of local eye-movement indicators.

Word spacing size	Tibetan proficiency	Regressive duration(ms)	Gaze duration(ms)	Total number of fixations	Total fixation duration(ms)
Normal	High proficiency	407.18 (327.62)	321.61 (183.60)	1.99 (1.34)	486.31 (328.36)
	Low proficiency	700.47 (568.79)	410.67 (248.52)	3.09 (2.01)	791.17 (523.08)
Half-width spaced	High proficiency	396.68 (277.13)	316.12 (177.52)	2.00 (1.33)	480.67 (312.10)
	Low proficiency	727.86 (631.41)	385.74 (226.04)	3.06 (2.07)	786.56 (535.47)
Full-width spaced	High proficiency	357.94 (302.87)	281.58 (139.49)	1.57 (1.17)	400.06 (269.50)
	Low proficiency	576.49 (530.86)	349.50 (209.14)	2.47 (1.91)	650.60 (484.54)

**Table 5:** Statistical analysis results of local eye-movement indicators.

Effects and contrasts	Statistic	Regressive duration (ms)	Gaze duration (ms)	Total number of fixations	Total fixation furation (ms)
Tibetan proficiency	*b*	−0.40	−0.2	−0.28	−0.45
	SE	0.08	0.04	0.07	0.09
	*t*	−5.13^***^	−4.67^***^	−4.11^***^	−5.03^***^
Space half-width vs. normal	*b*	0.01	−0.06	−0.01	−0.03
	SE	0.03	0.02	0.01	0.02
	*t*	0.40	−2.82^**^	−0.93	−1.43
Space: full-width vs. normal	*b*	−0.25	−0.14	−0.2	−0.25
	SE	0.03	0.02	0.01	0.02
	*t*	−7.34^***^	−7.18^***^	−13.07^***^	−12.02^***^
Space: half-width vs. full-width	*b*	0.27	0.09	0.18	0.22
	SE	0.03	0.02	0.01	0.02
	*t*	7.77^***^	4.39^***^	12.17^***^	10.59^***^
Proficiency × Space: half-width vs. normal	*b*	0.01	0.04	0.01	0.02
	SE	0.05	0.03	0.02	0.03
	*t*	0.02	1.51	0.65	0.80
Proficiency × Space: full-width vs. normal	*b*	0.13	0.04	0.04	0.07
	SE	0.05	0.03	0.02	0.03
	*t*	2.48^*^	1.5	2.17^*^	2.42^*^
Proficiency × Space: half-width vs. full-width	*b*	−0.13	−0.01	−0.03	−0.05
	SE	0.05	0.03	0.02	0.03
	*t*	−2.57^*^	−0.01	−1.52	−1.63

Note. ^***^ *p* < 0.001, ^**^ *p* < 0.01, ^*^ *p* < 0.05.

The main effect of Tibetan proficiency was significant. Specifically, compared to the low-proficiency group, the high-proficiency group exhibited shorter regressive duration (*b* = −0.40, SE = 0.08, *t* = −5.13, *p* < 0.001), shorter gaze duration (*b* = −0.20, SE = 0.04, *t* = −4.67, *p* < 0.001), and shorter total fixation duration (*b* = −0.45, SE = 0.09, *t* = −5.03, *p* < 0.001), as well as fewer fixations (*b* = −0.28, SE = 0.07, *t* = −4.11, *p* < 0.001).

The main effect of word spacing size was significant. Specifically, compared to the normal condition, the half-width spaced condition resulted in shorter gaze duration (*b* = −0.06, SE = 0.02, *t* = −2.82, *p* < 0.001). Compared to the normal condition, the full-width spaced condition exhibited shorter regressive duration (*b* = −0.25, SE = 0.03, *t* = −7.34, *p* < 0.001), shorter gaze duration (*b* = −0.14, SE = 0.02, *t* = −7.18, *p* < 0.001), shorter total fixation duration (*b* = −0.25, SE = 0.02, *t* = −12.02, *p* < 0.001), and fewer fixations (*b* = −0.20, SE = 0.01, *t* = −13.07, *p* < 0.001). Furthermore, compared to the full-width spaced condition, the half-width spaced condition led to longer regressive duration (*b* = 0.27, SE = 0.03, *t* = 7.77, *p* < 0.001), longer gaze duration (*b* = 0.09, SE = 0.02, *t* = 4.39, *p* < 0.001), longer total fixation duration (*b* = 0.22, SE = 0.02, *t* = 10.59, *p* < 0.001)., and a greater number of fixations (*b* = 0.18, SE = 0.01, *t* = 12.17, *p* < 0.001).

The interaction between Tibetan proficiency and word spacing size was significant. Specifically, in the contrast between full-width spaced and normal conditions, significant interactions were observed in regressive duration (*b* = 0.13, SE = 0.05, *t* = 2.48, *p* < 0.05), number of fixations (*b* = 0.04, SE = 0.02, *t* = 2.17, *p* < 0.05), and total fixation duration (*b* = 0.07, SE = 0.03, *t* = 2.42, *p* < 0.05). In the contrast between half-width spaced and full-width spaced conditions, a significant interaction was found only in regressive duration (*b* = −0.13, SE = 0.05, *t* = −2.57, *p* < 0.05). These results indicate that the impact of word spacing on reading is significantly modulated by Tibetan proficiency. Further simple effect analysis revealed that for the high-proficiency group, compared to the normal condition, the full-width spaced condition resulted in shorter regressive duration (*b* = −0.13, SE = 0.04, *t* = −3.54, *p* < 0.001), shorter total fixation duration (*b* = −0.19, SE = 0.02, *t* = −10.24, *p* < 0.001), and fewer fixations (*b* = −0.07, SE = 0.006, *t* = −11.52, *p* < 0.001); Compared to the half-width spaced condition, the full-width spaced condition also showed shorter regressive duration (*b* = −0.14, SE = 0.04, *t* = −3.79, *p* < 0.001). For the low-proficiency group, compared to the normal condition, the full-width spaced condition exhibited shorter regressive duration (*b* = −0.26, SE = 0.03, *t* = −7.57, *p* < 0.001), shorter total fixation duration (*b* = −0.24, SE = 0.02, *t* = −11.81, *p* < 0.001), and fewer fixations (*b* = −0.08, SE = 0.01, *t* = −12.62, *p* < 0.001); compared to the half-width spaced condition, the full-width spaced condition likewise resulted in shorter regressive duration (*b* = −0.27, SE = 0.03, *t* = −7.91, *p* < 0.001). While the performance of both high- and low-proficiency groups tended to be consistent across these indicators, the facilitative effect of the full-width spaced condition compared to the normal condition was more pronounced for the low-proficiency group: (1) the mean total fixation time decreased by 140.57 ms for the low-proficiency group and 86.25 ms for the high-proficiency group, representing a larger decrease of 54.32 ms in the former; (2) the mean regressive duration decreased by 123.98 ms for the low-proficiency group and 49.24 ms for the high-proficiency group, representing a larger decrease of 74.74 ms in the former; (3) the mean number of fixations decreased by 0.62 for the low-proficiency group and 0.42 for the high-proficiency group, representing a larger decrease of 0.2 in the former. Similarly, the significant interaction between half-width and full-width spaced conditions further confirmed that the full-width condition led to a more pronounced reduction in regressive duration for low-proficiency readers than for high-proficiency ones. In conclusion, the full-width spaced condition exerts a greater facilitative effect on lexical recognition for low-proficiency Tibetan readers.

Local analysis revealed a significant main effect of word spacing size. Specifically, compared to the normal condition, the half-width spaced condition resulted in shorter gaze duration. Furthermore, compared to both the normal and half-width spaced conditions, the full-width spaced condition led to shorter regressive duration, gaze duration, and total fixation duration, as well as fewer fixations. These findings indicate that inter-word spaces exert a facilitative effect on lexical recognition in Tibetan, and this facilitation increases as the spacing size increases. Additionally, a significant interaction between Tibetan proficiency and word spacing size was observed across three indicators: total fixation duration, regressive duration, and total number of fixations, suggesting that Tibetan proficiency modulates the word spacing effect. Further simple effect analysis demonstrated that full-width spaces provide a greater facilitative effect on lexical recognition for low-proficiency Tibetan readers.

## 4 Discussion

This study recruited students from Xizang University as participants and employed eye-tracking technology to investigate the following two issues by manipulating word spacing size in Tibetan texts and considering individual differences in Tibetan proficiency: (1) the effect of interword space size on the word segmentation effect during Tibetan reading; (2) the moderating role of Tibetan proficiency in this word segmentation effect. The experimental results indicated that: (1) word spaces significantly facilitate Tibetan text reading and lexical recognition, and this facilitative effect increases as the space size increases; (2) Tibetan proficiency moderates the spacing effect, such that full-width spaces provide a greater boost to lexical recognition for readers with lower Tibetan proficiency.

### 4.1 The effect of word spacing size on word segmentation in Tibetan reading

The results of the present study first supported Hypothesis H1: inserting interword spaces in Tibetan text significantly promotes reading and lexical recognition, and full-width spaces yield the strongest facilitative effect. This finding is consistent with existing evidence that Tibetan word boundaries facilitate saccade target selection and lexical recognition, and further reveals that this effect is moderated by word spacing size (Wang et al., 2024).

Notably, no significant differences were found among the three conditions (normal, half-width spaced, and full-width spaced) in total sentence reading time. This indicates that manually inserting interword spaces of different sizes did not significantly shorten or prolong the total time participants required to read the sentences. This result is similar to findings from Chinese reading research (Bai et al., 2008; Ma and Li, 2014). According to previous accounts, there may be a trade-off between the interference effect caused by unfamiliar text presentation and the facilitative effect provided by clear word boundaries (Bai et al., 2008). In the half-width and full-width spaced conditions, to isolate the pure effect of interword spacing, the Tibetan syllable delimiter (tsek) at word boundaries was removed and replaced by blank spaces. This manipulation disrupted readers' long-established reading habits and may have further increased processing load, thereby offsetting the facilitative effect of spaces to some extent. Furthermore, as a comprehensive indicator, total sentence reading time is simultaneously influenced by both early saccade planning and late integrative processing. Effects from different stages may statistically cancel each other out, which could be a major reason why total sentence reading time is relatively insensitive to changes in word spacing size.

Although no significant differences were observed in total reading time, various eye-movement measures indicate that different word spacing sizes facilitate Tibetan reading and word identification to varying degrees. Under the half-width space condition, readers exhibited larger mean saccade amplitudes and forward saccade amplitudes, alongside significantly shortened gaze durations. This suggests that half-width spaces may facilitate saccade planning and lexical information acquisition to some extent. However, the facilitative effect of full-width spaces was even more pronounced: compared to both the normal and half-width space conditions, full-width spaces not only further reduced mean fixation durations and increased saccade amplitudes overall but also decreased regression time, gaze duration, and total fixation time in local processing, while significantly reducing the number of fixations. The above analysis demonstrates that because half-width spaces provide relatively weak segmentation information, their facilitative effect is limited. In contrast, the clear physical boundaries provided by full-width spaces not only optimize parafoveal saccade target selection but also effectively reduce the cognitive load associated with word identification and late-stage semantic integration. In summary, word spacing significantly facilitates Tibetan text reading and word identification, and this facilitative effect strengthens as word spacing size increases.

It is also worth noting that inserting spaces increased the physical length of the sentences, which may have influenced some of the observed eye-movement effects. The larger saccade amplitudes in the spaced conditions may partly reflect the greater distance readers needed to traverse, rather than improved saccade planning alone. The increased display length may also have offset some of the processing benefits of clearer word boundaries, contributing to the null effect on total reading time. However, physical lengthening alone cannot readily explain the reductions in fixation duration, regression duration, gaze duration, and total fixation time, particularly under the full-width condition. Taken together, these changes, which are not readily attributable to physical lengthening, provide evidence that word spacing facilitated lexical processing and sentence comprehension.

Regarding the effect of word spacing size, the present study demonstrates cross-linguistic differences compared with Chinese reading. Previous research has found that half-character spaces exerted no significant influence on reading performance, whereas full-character spaces disrupted the visual coherence of Chinese text and inhibited reading (Liang, 2010). In the present study, however, full-width spaces showed the strongest facilitative effect in Tibetan reading. This divergence from Chinese reading may arise from variations in orthographic structure. As a logographic writing system, Chinese provides abundant visual and semantic cues within characters, so readers rely less on external physical segmentation cues (Li, 2020). In contrast, Tibetan characters are organized around a root consonant, with letters attached as prefixes, suffixes, or stacked vertically to form a complex spatial structure, which easily leads to visual crowding and increases word segmentation difficulty (Bai et al., 2017; Gao et al., 2015). This orthographic interpretation is further supported by evidence from Japanese reading. Interword spacing facilitates word identification and eye-movement guidance in text written entirely in Hiragana, whereas it provides no comparable benefit in mixed Kanji-Hiragana text, in which visually salient Kanji characters provide effective segmentation cues (Sainio et al., 2007). Taken together, the current results and the Japanese findings converge on the same principle, which is also consistent with Slattery (2013) claim: the higher the inherent segmentation difficulty of a text, the more benefits readers obtain from clear external physical segmentation cues.

Therefore, Tibetan reading relies more on the physical boundary cues provided by full-width spaces to reduce visual crowding and compensate for insufficient internal segmentation information, thus showing a unique facilitative advantage.

In summary, word spacing significantly facilitates Tibetan text reading and word recognition, and this facilitative effect strengthens as word spacing size increases. Although, no significant differences were observed in total reading time, eye-movement measures indicate that full-width spaces yield the optimal facilitative effect. This pattern also reveals a cross-linguistic discrepancy compared to Chinese reading.

### 4.2 The moderating role of Tibetan proficiency in the word spacing effect

The results of this study further validated Hypothesis H2: Tibetan proficiency plays a significant moderating role in the process by which word spacing size affects Tibetan reading, with the facilitative effect of full-width spaces being more prominent for readers with lower Tibetan proficiency during word recognition. This finding is highly consistent with existing research, suggesting that the moderating mechanism of language proficiency within the word segmentation effect possesses cross-linguistic stability.

The local analysis results reveal a significant moderating effect of language proficiency. Under the full-width spaced condition, the reductions in regressive duration, total fixation duration, and number of fixations were greater for low-proficiency readers than for high-proficiency readers. This indicates that the effect of full-width spaces in reducing cognitive load is more significant for readers with lower Tibetan proficiency. This discrepancy arises because low-proficiency readers possess weaker internal word segmentation skills and rely more heavily on clear external visual boundary cues to assist in lexical recognition and semantic integration. Due to the lack of natural word boundaries in Tibetan, low-proficiency readers must invest substantial cognitive resources in word segmentation under the normal condition. The introduction of full-width spaces provides explicit physical word boundaries, effectively reducing the segmentation burden and decreasing unnecessary regressions and repetitive processing, thereby significantly enhancing the efficiency of reading integration. This finding is highly consistent with the interactive-compensatory model of reading: when readers exhibit deficits in processing levels such as word segmentation, external visual cues play a greater compensatory role (Stanovich, 1980).

Notably, high-proficiency readers also exhibited significant reductions in regressive duration and total fixation duration under the full-width spaced condition, suggesting that clear external word boundaries can still alleviate their cognitive processing load to some extent. However, because high-proficiency readers themselves possess efficient word segmentation abilities and have a lower demand for external spaces, their magnitude of facilitation was markedly smaller than that of low-proficiency readers. In other words, high-proficiency readers are capable of completing reading tasks relatively fluently even under the normal condition.

These results are consistent with classic findings in the fields of reading and word segmentation. Specifically, relevant studies indicated that less skilled readers rely more heavily on external spatial boundaries to assist in eye-movement control and word identification than skilled readers (Shen et al., 2010). Further, research confirmed that word spacing more significantly improves the processing efficiency of weaker readers, compensating for their lack of internal automatic word segmentation skills (Yan et al., 2012). This mechanism also applies to second-language Chinese reading. A relevant meta-analysis has demonstrated a stronger overall word-spacing effect among international students and learners with lower Chinese proficiency (Yu and Ren, 2015). Extending this conclusion to the Tibetan–Chinese bilingual context, recent research has shown that individuals with lower language proficiency gain greater reading benefits from explicit external word segmentation cues Gao L. et al. (2024).

In summary, Tibetan proficiency significantly moderates the spacing effect, such that full-width spaces yield a stronger facilitative effect for readers with lower Tibetan proficiency, whereas high-proficiency readers exhibit less dependence on external word segmentation cues.

## 5 Conclusion

Based on the findings of this study, the following conclusions can be drawn:(1) Word spacing significantly facilitates Tibetan text reading and lexical recognition, and this facilitative effect strengthens as the word spacing size increases. (2) Tibetan proficiency moderates the word spacing effect, such that full-width spacing yields a stronger facilitative effect on lexical recognition for low-proficiency readers.

## Data Availability

The original contributions presented in the study are included in the article/supplementary material, further inquiries can be directed to the corresponding authors.
